# Revisiting the Randomized Controlled Trial

**DOI:** 10.12669/pjms.333.13221

**Published:** 2017

**Authors:** Hassam Zulfiqar

Randomized controlled trial (RCT) is generally considered to be one of the strongest study designs because the results it generates, have minimum bias and avoids confounders by virtue of the randomization and blinding technique which it employs. It has the potential to generate a Level B evidence on its own or even a Level A evidence when a pooled meta-analysis or a systematic review of several RCTs is conducted. The results a RCT generates occupy the top of hierarchy of evidence and if the trial is a large multicenter placebo controlled double blind one, people will have a hard time critically appraising it as it’s; statistically speaking, the strongest possible study design.

The inception of RCT dates back to World War II when in an effort to impose an objective and scientific discipline onto the extraordinary postwar expansion of medical research, the components of the double-blind RCT were adopted and coalesced together for the first time.^1^ These included blind assessment (usually meaning a placebo control), random assignment to comparable groups, and inferential statistics as a surrogate for determinism.^2^ Back then, Placebo was not considered to offer much help in bringing a measureable change in objectively recordable outcome variables or in improving patient’s health in general. On the contrary, Physicians used it routinely in their practice as for example; in 1807, Thomas Jefferson (1743–1826) penned a description of what he called the “pious fraud” and noted that, “one of the most successful physicians I have ever known has assured me that he used more bread pills, drops of colored water, and powders of hickory ashes, than of all other medicines put together”.^3^

It was not until late 1955 that for the first time someone elucidated that placebo could have healing potential with Henry K. Beecher publishing his most famous work “The Powerful Placebo”^4^ in JAMA highlighting this mysterious powerful force never described before. It was soon to become the standard control group of the randomised controlled trial. Instead of an inert sham given to individual patients, the placebo became the emblem for all the healing occurring in the disguised “no- treatment” arm of an RCT. The “placebo effect” encompassed all “nonspecific effects” that did not depend on the treatment in the active arm. The “powerful placebo” became a hodge-podge of nonlinear, difficult to quantify, remnants collected under the rubric of the dummy control of an RCT. Anything that threatened the fastidious detection of a predictable cause and effect outcome was conveniently disposed of in a repository labelled the “placebo effect”. This new concept of placebo was much larger both in meaning and power than its predecessor.^3^

Soon Randomized controlled trial became the standard of medical research and placebo became its integral component. Any new drug which came into the market had to undergo rigorous experimentation under the umbrella of randomised controlled trial and the drug had to perform better than the placebo arm in order to be considered significantly efficacious.

Though Beecher gave us a very rigorous way of experimentation but all his work was backed by an audacious yet simple assumption which the medical community ignored altogether. The rationale behind this was the ‘additive’ model which was first described by Beecher in his influential explicit assumption of an additive relation between placebo and drug effects^5^, ‘The placebo effect of active drugs is masked by their active effects… The total ‘drug’ effect is equal to its ‘active’ effect plus its placebo effect’ (quotes in the original).^4^ It assumes that the placebo effect is constant and additive in nature. The improvement seen in the experimental arm of RCT is simple summation of that seen in the placebo control arm and the improvement caused by the intervention itself. This assumption is a big one yet randomised, double-blind, placebo-controlled clinical trials continue to be considered as the gold standard to demonstrate the clinical efficacy of any drug or intervention.

Time and time again, numerous studies have shown that the model of additivity doesn’t necessarily holds true. Placebo effects are not supposed to be a constant phenomenon rather they depend on various neurobiological, genetic and epigenetic factors and hence vary from person to person. Moreover placebo can’t be attributed to the dummy pill alone rather it’s a collective healing effect which results from empathy, doctor-patient interaction and the ritual of medicine itself. Medicine has traditionally used placebos as a tool to challenge, debunk, and discard ineffective and harmful treatments. But placebo effects are another story; they are not bogus^6^ rather placebo is a complex neurobiological phenomenon and hence the assumption made by beecher model of additivity is way too simplistic. Given that the evaluation of drug treatments in RCTs is based on the assumption of additivity, its violation has far-reaching consequences. Therefore there is a need for novel study designs enabling researchers to consider the complex interplay of drug-specific and unspecific effects as noted recently by Kube et al.^7^
